# Gold nanoparticles (AuNPs) impair LPS-driven immune responses by promoting a tolerogenic-like dendritic cell phenotype with altered endosomal structures†

**DOI:** 10.1039/d0nr09153g

**Published:** 2021-04-15

**Authors:** Sara Michelini, Francesco Barbero, Alessandra Prinelli, Philip Steiner, Richard Weiss, Thomas Verwanger, Ancuela Andosch, Ursula Lütz-Meindl, Victor F. Puntes, Damjana Drobne, Albert Duschl, Jutta Horejs-Hoeck

**Affiliations:** Department of Biosciences, Paris-Lodron University Salzburg Hellbrunner Str. 34 5020 Salzburg Austria jutta.horejs_hoeck@sbg.ac.at; Insitut Català de Nanosciència i Nanotecnologia (ICN2) UAB Campus Bellaterra Barcelona 08193 Spain; AvantiCell Science Ltd Gibbsyard Building Auchincruive Ayr KA6 5HW UK; Biotechnical Faculty, University of Ljubljana Večna pot 111 1000 Ljubljana Slovenia

## Abstract

Dendritic cells (DCs) shape immune responses by influencing T-cell activation. Thus, they are considered both an interesting model for studying nano-immune interactions and a promising target for nano-based biomedical applications. However, the accentuated ability of nanoparticles (NPs) to interact with biomolecules may have an impact on DC function that poses an unexpected risk of unbalanced immune reactions. Here, we investigated the potential effects of gold nanoparticles (AuNPs) on DC function and the consequences for effector and memory T-cell responses in the presence of the microbial inflammatory stimulus lipopolysaccharide (LPS). Overall, we found that, in the absence of LPS, none of the tested NPs induced a DC response. However, whereas 4-, 8-, and 11 nm AuNPs did not modulate LPS-dependent immune responses, 26 nm AuNPs shifted the phenotype of LPS-activated DCs toward a tolerogenic state, characterized by downregulation of CD86, IL-12 and IL-27, upregulation of ILT3, and induction of class E compartments. Moreover, this DC phenotype was less proficient in promoting Th1 activation and central memory T-cell proliferation. Taken together, these findings support the perception that AuNPs are safe under homeostatic conditions; however, particular care should be taken in patients experiencing a current infection or disorders of the immune system.

## Abstract 2

This study shows that gold nanoparticles promote the differentiation of dendritic cells to a tolerogenic-like phenotype, affecting their ability to induce antibacterial immune responses mediated by Th1 cells and to activate central memory T cells.

## Graphical abstract

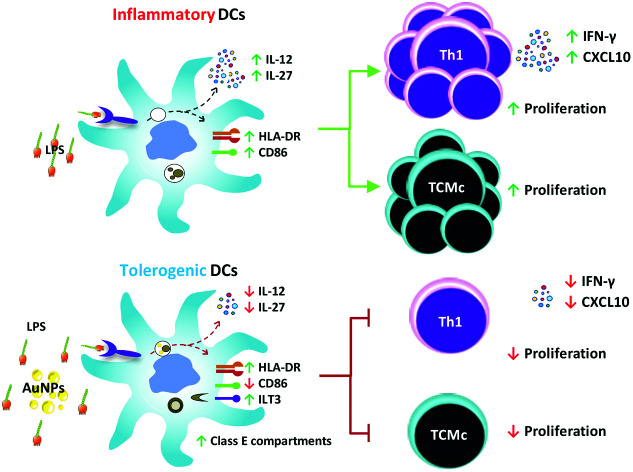

## 1. Introduction

The human immune system can be divided in two branches: the innate and the adaptive immune system. The innate immune system represents the first line of defense against pathogens, damaged cells and non-self-objects (*e.g.* nanoparticles and microbes) and is characterized by a quick and conserved set of responses carried out by innate immune cells.^1^

Innate immune responses are usually followed by activation of the adaptive immune system, which is capable of mounting a pathogen-specific response mediated by T cells and B cells.

Antigen presenting cells (APCs) act at the interface between the innate and the adaptive immune systems, with dendritic cells (DCs) being considered the most important type of APC. APCs are usually located within tissues and are equipped with a broad spectrum of receptors which enables them to sense the presence of pathogens by recognizing conserved molecular patterns on their surface.^2,3^ One of these patterns is the endotoxin lipopolysaccharide (LPS), located on the outer membrane of Gram-negative bacteria and recognized by a receptor complex composed of Toll-like receptor 4 (TLR4), MD2 and CD14 on the DC surface.^4^ Upon pathogen recognition, DCs become activated and begin a maturation process characterized by the upregulation of co-stimulatory surface molecules, including CD86 and HLA-DR, and the release of specific cytokines. This maturation process enables the DCs to activate naïve T cells, thereby initiating adaptive immune responses. Based on signals provided by DCs (*e.g.* cytokines and surface receptors) during the DC–T-cell interaction, naïve T cells can polarize into pathogen-specific T helper (Th) cell subsets, which are responsible for coordinating the elimination of the invading pathogen. Among the different Th cell subsets, Th1 cells have the task of eliminating intracellular bacteria and viruses, whereas Th2 cells protect the body against extracellular parasites. Th17 cells orchestrate the fight against extracellular bacteria or fungi, while regulatory T cells (Tregs) have the crucial role of suppressing excessive inflammation and autoimmunity.^5–11^ However, unbalanced Th cell polarization and activation is often linked to immune-related pathologies.^12,13^ Interestingly, each of these Th cell subsets, also known as effector T cells, can be identified using specific molecular markers such as cytokines and surface markers.^11^ While many effector T cells die during pathogen clearance, a specific Th cell population termed “memory cells” remains poised for years and decades and is crucial for establishing long-term protection.^14,15^

Because of their central role in shaping immune responses, DCs are considered an important model to investigate potential immunomodulatory properties of nanoparticles (NPs). Upon interaction of NPs with biomolecules, a biomolecular corona is formed, which has a significant influence on the fate and effects of NPs in tissues and cells.^16–19^ The interactions between NPs and cells can be intentionally exploited in medical applications, like cancer treatment or diagnosis;^20,21^ however, unintended interactions can have undesired effects.

In this context, gold nanoparticles (AuNPs) have attracted particular attention due to their stability, biocompatibility, and interesting optical properties, which make them not only suitable for different applications in biomedicine, but also a widely used “model particle” for studying the molecular basis of nano-immune interactions.^21,22^

Therefore, the aim of this work was to study the potential effects of AuNPs on the ability of DCs to promote effector and memory T-cell activation and proliferation during LPS-induced immune responses.

To achieve this goal, we first investigated the potential immunomodulatory properties of endotoxin free-AuNPs of different sizes (4, 8, 11 and 26 nm) in terms of cytokine secretion (IL-12, IL-27) and surface marker expression (CD86, HLA-DR, ILT3) in LPS-treated human monocyte-derived DCs (moDCs). Next, we analyzed AuNP-induced ultrastructure and morphological changes, which are of special interest because internalized NPs may affect the endosomal machinery of mature DCs known as “endosomal sorting system required for transport (ESCRT)”, which is involved in several processes relevant for cell-mediated immune responses.^23–25^

Subsequently, to investigate whether the identified changes in DC phenotype can alter T-cell polarization, we performed co-culture experiments of AuNP-treated, LPS-stimulated DCs with CD4^+^ Th cells and evaluated the production of Th1 cell-specific cytokines.

Finally, to study the effects of NPs in a more complex model, we incubated human peripheral blood mononuclear cells (PBMCs) with AuNPs and/or LPS and assessed both effector and memory T-cell responses using an advanced 13-color flow cytometry panel.

## 2. Experimental section

### 2.1 AuNP synthesis

Sodium citrate-stabilized gold nanoparticles (AuNPs) were synthesized using two kinetically controlled seeded growth methods as described by Bastús *et al.*^26^ and by Piella *et al.* (for the 4 nm particles)^27^ using tetrachloroauric(iii) acid trihydrate (99.9% purity) and sodium citrate tribasic dihydrate (≥99%) (SC) purchased from Sigma-Aldrich.

Since innate immune cells are extremely sensitive to endotoxin contamination, we applied an “LPS-free” synthesis protocol to prevent unspecific cell activation in our experiments. Briefly, the reagent solutions for the synthesis were prepared with endotoxin-free material and with LPS-free water (Cape Cod Incorporated) under a flow hood. Disposable plastic materials and syringe needles were purchased LPS-free (B. Braun), lab glassware was wrapped with aluminum foil and depyrogenated in a lab oven at 200 °C overnight, and rubber stoppers and stir bars were cleaned with ethanol and left to dry in the flow hood. To quantify potential residual LPS contamination, we performed both an NF-κB-luc reporter gene assay and an EndoLISA assay.

### 2.2 AuNP physicochemical characterization

NP post-synthesis characterization and analysis of protein corona formation in relevant culture medium were performed using four different techniques. The use of such techniques for NP characterization was shown to be reliable in previous studies.^26,27^

#### Electron microscopy

Size and shape of the AuNPs were assessed *via* Scanning Electron Microscopy using an FEI Magellan XHR SEM in transmission mode (STEM) operated at 20 kV and an FEI Tecnai G2 F20 S-TWIN HR(S)TEM operated at an accelerated voltage of 200 kV. After synthesis, 2 μL of the sample were drop-casted on a carbon-coated copper TEM grid and dried at room temperature (RT) using a vacuum drier. To avoid handling-dependent aggregation during sample preparation, AuNPs were functionalized by adding 11-mercaptoundecanoic acid (MUA) (Sigma-Aldrich) at a final concentration of 0.1 mM. The solution was then left for 2 h at RT under slow-stirring conditions; subsequently, NPs were purified from the MUA excess by centrifugation. After image collection, particles were analyzed using ImageJ to obtain reliable size-distribution data.

#### UV-vis spectroscopy

A Shimadzu UV-2400 spectrophotometer was used to acquire the UV-vis spectra. Spectral acquisition was performed at RT in the 300–750 nm range using disposable plastic cuvettes. MilliQ water and complete culture medium were used as a reference.

#### Size and zeta potential measurements

The hydrodynamic diameter and *Z* potential of AuNPs both in SC and after incubation in moDC medium were assessed at 37 °C using a Malvern Zetasizer Nano ZS by Dynamic Light Scattering (DLS), and Laser Doppler Velocimetry (LDV). Diameters were reported as a distribution by intensity calculated by non-negative least squares (NNLS) analysis. *Z* potential is not an intrinsic value but depends on the conductivity of the solution in which NPs are dispersed. Consequently, to compare the *Z* potential of different samples, their conductivity has to be similar. Thus, after exposure to the medium the AuNPs were purified by centrifugation and resuspended in SC (2.2 mM) to obtain a final conductivity similar to that of the pristine AuNPs (about 0.7 mS cm^−1^).

#### AuNP behavior in culture medium

To evaluate the stability of our NPs in moDC medium and to assess the formation of the protein corona, we incubated the particles for 1, 24 and 48 h, 5 × 10^11^ AuNPs per well with 900 μL of complete culture medium [moDC medium: RPMI-1640 (Sigma) supplemented with 10% heat-inactivated (i.a.) pure fetal bovine serum (FBS) (PAA) (Thermo-Fisher), 2 mM l-glutamine (Sigma), 100 U mL^−1^, penicillin/streptomycin (Sigma), 50 μM β-mercaptoethanol (Gibco Laboratories)]. Differences in volume between the AuNP batches were compensated by the addition of LPS-free SC 2.2 mM up to a final volume of 1.2 mL. Since the absorption spectrum generated by the pH-sensing molecule phenol red is comparable to the one obtained for AuNPs, we used a phenol red-free RPMI [RPMI-1640 R7509]. Sodium citrate, moDC medium and AuNPs in SC were used as controls.

### 2.3 Cell culture conditions

All cells were cultured at 37 °C, 5% CO_2_ and 95% humidity. The incubation time of nanoparticles and cells was estimated on the basis of the findings of Casals *et al.*,^28^ who showed that protein corona formation reaches a stability point (hard corona) after approximately 48 h of incubation in culture medium.

In order to exclude potential side effects of endotoxin contamination in AuNPs, we used a concentration of AuNPs which was insufficient to activate immune cells. This concentration was calculated on the basis of the endotoxin content measured by the EndoLISA assay. Since every batch possessed different levels of contamination, we used the highest possible amount of 26 nm AuNPs as a reference (5 × 10^11^ NPs per well) and equalized the concentration of the other batches in terms of particle number. Because of the different sizes of the used AuNPs, maintaining the number of particles applied on cells simultaneously increased the amounts of gold and surface area in a way that is directly proportional to the particle diameter. Detailed information on corresponding concentrations of NPs in terms of μg mL^−1^ or surface area per μL can be calculated using Table 1 assuming a 100% spherical shape. Since the synthetic method used in this paper generates particles that are more and more diluted after every growing step, in order to apply 5 × 10^11^ NPs per well we added different volumes of AuNP stock solution. The different volumes were then equalized by the addition of SC 2.2 mM, which is used as a stabilizer during the NP synthesis. LPS concentration was based on previous studies.^29^

**Table 1:** AuNP post-synthesis characterization

Characteristics	Batches			
Name	4	8	11	26
Material	Au	Au	Au	Au
Stabilizer	Sodium citrate	Sodium citrate	Sodium citrate	Sodium citrate
Diameter by STEM [nm]	4.0 ± 0.6	8.8 ± 1.5	11.8 ± 0.8	26.4 ± 3.0
Hydrodynamic Diameter [nm]	5.6 ± 1.4	12.8 ± 3.0	15.1 ± 3.7	36.8 ± 14.1
Absorption peak [nm]	504	519	516	523
*Z* potential [mV]	Not detected.	−49.3 ± 5.0	−47.0 ± 5.0	−35.6 ± 0.6
Concentration [NPs per ml]	5.0 × 10^13^	5.0 × 10^12^	6.0 × 10^12^	1.7 × 10^12^
Au concentration [mM]	0.167	0.166	0.500	1.650
Au concentration [mg mL^−1^]	0.033	0.033	0.100	0.325
Total surface area [nm^2^ mL^−1^]	2.5 × 10^15^	1.2 × 10^15^	2.6 × 10^15^	3.8 × 10^15^

### 2.4 Quantification of LPS-contamination

Two different methods were used to detect LPS contamination.

#### EndoLISA (Hyglos Gmbh)

EndoLISA was used for quantitative determination of endotoxin contamination of AuNP preparations, according to the manufacturer's recommendations. Samples were diluted 1 : 2 in LPS-free water and the binding step was carried out for 18 h. Acquisition was performed on a microplate reader (Tecan Infinite M200Pro) with a fixed gain of 100. Analysis was performed using a 4-Parameters Logistic Regression Model. LPS contamination was then expressed in EU mL^−1^ (EU, endotoxin units).

#### NF-κB-luc TLR4 reporter gene assay

As described by Schwarz *et al.*,^30^ HEK293 cells (mycoplasma negative, culture passage 5–15) were seeded in DMEM [DMEM (Sigma-Aldrich) supplemented with 10% FBS, 2 mM MEM non-essential amino acids (PAA) (Sigma-Aldrich), 2 mM l-glutamine, 100 U mL^−1^ penicillin/streptomycin] at a final concentration of 1.5 × 10^5^ cells per mL and incubated for 24 h. On day 2, cells were transfected with both a reporter plasmid containing the NF-κB transcription factor linked to a luciferase reporter gene, and a mix of 3 plasmids (TLR4, MD2 and CD14) encoding the LPS receptor components. After 24 h, supernatants were discarded and substituted with 900 μL of fresh medium. Cells were then stimulated by the addition of LPS 30 pg mL^−1^ (*E. coli* lipopolysaccharide (LPS) 055:B5 (Sigma-Aldrich)), AuNPs of different sizes (5 × 10^11^ NPs per well), LPS plus AuNPs, or left untreated. Volume differences were compensated by adding PBS to a final volume of 1.2 mL. On day 4, supernatants were discarded, cells were lysed and lysates were read by a Tecan Infinite M200Pro instrument. To exclude AuNP interference in signal absorbance, data are expressed in the form of a ratio between LPS or AuNPs + LPS and the corresponding controls, untreated cells or AuNPs.

### 2.5 Generation of human monocyte-derived dendritic cells (moDCs)

PBMCs were isolated from buffy coats of donors by density gradient centrifugation using histopaque-1077 (Sigma) as described by Posselt *et al.*^31^ The buffy coats used in this study were kindly provided by the blood bank of Salzburg, Austria. According to Austrian regulations, no informed consent is required if blood cells derived from anonymous healthy donors, discarded after plasmapheresis (buffy coats), are used. Therefore, no additional approval by the national ethics committee was required. Monocytes were then purified from PBMCs using the CD14^+^ MicroBeads UltraPure human kit (Miltenyi Biotec) according to the manufacturer's instruction. Subsequently, cells were cultured for 6 days in moDC medium (2.5 × 10^5^ cells per mL, supplemented with 50 ng mL^−1^ GM-CSF and 50 ng mL^−1^ IL-4 (a generous gift from Novartis)). After 48 h, monocytes were fed 1 vol. medium containing fresh IL-4 and GM-CSF at a final concentration of 50 ng mL^−1^. To assess the efficacy of the differentiation protocol, DC and monocyte lineage marker expression and morphology were assessed by both flow cytometry and differential staining (Diff–Quik; Medion Diagnostics) following Pickl work.^32^ Residual CD14^−^ CD1a^−^ cells, most likely polymorphonuclear granulocytes (PMNGs), which can be observed after monocyte isolation, are characterized by a limited lifespan and will therefore die during the process of moDC differentiation.

### 2.6 Cytotoxicity and viability assessment

1 × 10^5^ moDCs were plated in 900 μL of DC medium and stimulated for 48 h either with LPS (30 ng mL^−1^, final concentration), different sizes of AuNPs (5 × 10^11^ NPs per well) or left untreated. Volume differences were compensated with sterile-filtered LPS-free SC 2.2 mM to a final volume of 1.2 mL. To detect toxicity, two methods were used.

#### LDH assay (Promega)

Cytotoxicity was assessed by quantification of lactate dehydrogenase (LDH) release in 90 μL of cell supernatant using a “CytoTox 96® Non-Radioactive Cytotoxicity assay kit” following the manufacturer's recommendations. As a positive control, cells were lysed with 0.10% (v/v) Triton X-100 for 30 min prior to harvesting to achieve maximum LDH release.

#### CellTiter-blue (CTB) (Promega)

Cell viability was assessed *via* CTB assay following the manufacturer's instructions. After 1 h of incubation, supernatants were analyzed. Cells treated with Triton X-100 served as a control for low viability. A microplate reader (Tecan Infinite M200Pro) was used to record sample absorbance (LDH) and fluorescence emission (CTB) following the manufacturer's instructions.

### 2.7 Impact of AuNPs on LPS-stimulated moDCs

To assess the potential impact of AuNPs of different sizes on LPS-induced immune responses, 1 × 10^5^ DCs were plated in 900 μL of DC medium and stimulated for 48 h either with LPS (30 ng mL^−1^, final concentration), AuNPs alone (5 × 10^11^ NPs per well), LPS plus AuNPs, or left untreated. The NP dose (5 × 10^11^ NPs per well) was chosen because it is the highest concentration that can be added without inducing cell activation by LPS contamination and without diluting the cell culture medium to an extent that could result in reduced cell viability and NP aggregation.

Volume differences were compensated with SC 2.2 mM up to a final volume of 1.2 mL. After 48 h, supernatants were collected and tested for IL-6, IL-8/CXCL8, IL-12, TNF-α (Peprotech) and IL-27 (R&D) release by ELISA. Cell surface markers and cell structures were analyzed *via* flow cytometry or TEM.

### 2.8 CD4^+^ T-cell isolation and co-culture with moDCs

DCs were stimulated as described before. Briefly, 0.25 × 10^5^ DCs per well were cultured for 48 h in 225 μL of moDC medium supplemented with the previously stated stimuli in a 48-well plate (LPS 30 ng mL^−1^ final concentration, AuNPs 1.25 × 10^11^ NPs per well, LPS + AuNPs). Untreated cells served as a negative control. Again, volume differences were compensated with SC up to a final volume of 300 μL. After 48 h, allogeneic CD4^+^ T cells were isolated from buffy coats using a Human CD4^+^ T cell Isolation Kit from Miltenyi Biotec, added to moDCs at a ratio of 10 : 1 in 300 μL of moDC medium supplemented with IL-2 (100 U mL^−1^) and co-cultured for 6 days. After 72 h, IL-2 50 U mL^−1^ was added to each well to promote T-cell survival. On day 6, supernatants were harvested and tested for cytokine release by ELISA (Peprotech) and LEGENDplex.

### 2.9 Generation and culture of human PBMCs

PBMCs were isolated from buffy coats as described in section 2.5 and seeded in a 24-well plate at a concentration of 1 × 10^6^ in 900 μL, and stimulated for 6 days either with LPS (30 ng mL^−1^, final concentration), AuNPs of 4-, 8-, 11- and 26 nm (5 × 10^11^ NPs per well), LPS plus AuNPs, or left untreated. The final volume was then equalized by the addition of SC 2.2 mM up to a final volume of 1.2 mL. Subsequently, supernatants were harvested and analyzed *via* ELISA (R&D), while cells were stained and analyzed using a 13-color flow cytometry panel. The number of cells used in this experiment was calculated based on the composition of PBMCs in healthy individuals, which presents an average amount of APCs (monocytes plus DCs) ranging between 11% and 22% of the total population. By using 1 × 10^6^ cells per well, we guaranteed the presence of at least 1 × 10^5^ APCs in the preparation, which is in line with the experiments performed with DCs (see sections 2.6 and 2.7).^33^

### 2.10 Flow cytometry

Surface marker expression on DCs was assessed *via* flow cytometry by staining the cells with α-HLA-DR APC, Fixable Viability Dye eFluor506, α-CD1a BV421, α-CD86 PE (eBioscience), α-CD14 PerCP-Cy5.5 (BD Bioscience) and α-ILT3 PerCP-Cy5.5 (Biolegend). Samples were subsequently acquired using a FACS Canto II flow cytometer (BD Biosciences). The shown MFI data were collected after exclusion of doublets and dead cells.

Surface marker expression on PBMCs was assessed *via* flow cytometry using a 13-color flow-cytometry panel generated by using the Optimized Multicolor Immunofluorescence Panel-030 (OMIP-030)^34^ as a reference: CD4 BV510, CD127 AlexaFluor 700, CD8a PE-Cy5, CD3 PerCP-Cy5.5, CD45RA BV605, CD25 PE, CD20 APC-Cy7, CD194 PE-Cy7, CD197 PE/Dazzle594 (Biolegend), CCR10 APC (BD Biosciences), Ki-67 BV650, CD196 BB151, CD183 BV421 (BD Horizon) Fixable Viability Dye APC-Cy7 (eBioscience). Samples were acquired using a Cytoflex S flow cytometer (Beckman Coulter) and then analyzed using the gating strategy summarized in ESI Fig. 5.†

### 2.11 LEGENDplex™

Supernatants were centrifuged in 96-well V-bottom plates for 10 min at 1000 g to eliminate NPs which might interfere with the assay. The release of cytokines was assessed using the LEGENDplex™ Human Th Panel (13-plex) (Biolegend) kit, according to the manufacturer's recommendations. Sample acquisition was performed using a Cytoflex S flow cytometer (Beckman Coulter), and LEGENDplex v8.0 Analyst Software was used for data evaluation.

### 2.12 Transmission electron microscopic (TEM) preparation of DCs

After stimulation, moDCs were transferred into sample holders for subsequent high-pressure freeze fixation (HPF). HPF was carried out in a Leica Empact HPF device (Leica Microsystems), with a cooling rate of approximately 12 000 °C s^−1^ and a pressure value of at least 2040 bar. The cryo-substitution was transacted with a Leica EM AFS (Leica Microsystems) at defined cycles.^35^ The substitution medium contained 2% osmium tetroxide (OsO_4_) and 0.05% uranyl acetate in anhydrous acetone. After the cryo-substitution, the samples were washed three times with anhydrous acetone and propylene oxide and were then embedded into epoxy resin (medium grade; Agar Scientific). The embedding of samples was carried out in Beem® capsules (Agar Scientific) to ensure that moDC samples would sink and gather at the narrow bottom of the capsules for further preparation. Afterwards, samples were polymerized at +70 °C for 24 h. Ultra-thin sectioning (∼70 nm) was performed with a Leica UC7 Ultramicrotome (Leica Microsystems) and sections were collected on Formvar-coated copper grids. TEM preparation was conducted as in previous studies and adapted for DCs.^35,36^

### 2.13 Transmission electron microscopic (TEM) imaging of DCs

TEM imaging of DCs was carried out by using a LEO 912 AB TEM with an in-column Omega energy filter (Zeiss) at 80 kV accelerating voltage. TEM images were recorded with a Tröndle TRS Sharp Eye bottom-mounted 2 K CCD camera (Tröndle), filtered at zero energy loss. The TEM implementation and recording process were controlled by iTEM 5.0 software (Olympus).

### 2.14 Statistical analysis

Statistical analyses were performed using GraphPad Prism Software Version 6.01. *P* values for multiple groups were calculated *via* repeated-measures ANOVA with a Tukey's *post-hoc* test. Data are shown as mean + SD. The Geisser-Greenhouse correction was not applied. *P*-values < 0.05 were considered statistically significant (*p < 0.05, **p < 0.01, ***p < 0.001, ****p < 0.0001).

### 2.15 Reported information about the experiments

The experiments were conducted and reported following MIRIBEL guidelines.^37^

## 3. Results and discussion

### 3.1 AuNP characterization both after synthesis and in relevant culture medium

As reported by other authors, the majority of immune responses to nanomaterials (NMs) are characterized by an adaptation process resulting in silent elimination of the NM or transient inflammation followed by full resolution.^1^ However, despite being regarded as reasonably safe when administered in therapeutic doses and under homeostatic conditions, some NMs may have deleterious effects in cases where there is a less balanced immune system, *e.g.* during acute inflammation. Due to their elevated energy potential, NPs are highly reactive objects that constantly interact with other molecules in order to achieve a more stable thermodynamic status. This might result in the binding and functional alteration of biomolecules and signaling pathways which play a critical role in defining the outcome of an immune response, ultimately leading to the establishment of undesired immunological side effects.^17^ Therefore, evaluation of immunomodulatory effects of NMs is crucial to ensure patient safety.

Since the reactivity of NPs is strongly influenced by their composition, size, charge, shape, and aggregation status, it is of outmost importance to carry out a comprehensive characterization of NMs used in biological studies. In this work in particular, characterization of our gold nanoparticles was performed both after synthesis and in relevant culture conditions following MIRIBEL (Minimum Information Reporting in Bio-Nano Experimental Literature) recommendations.^37^

**Fig. 1:**
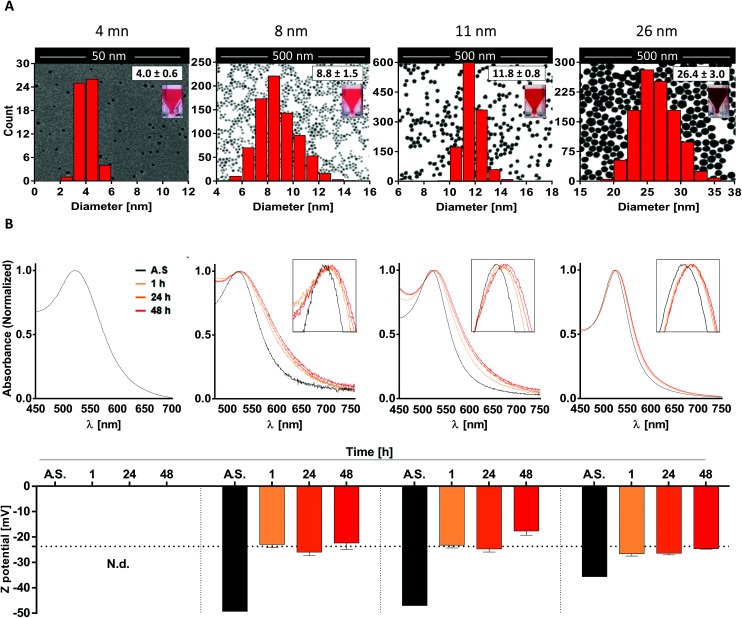
AuNP post-synthesis characterization and behavior in culture medium. (A) Representative TEM images of AuNP samples overlaid with both the corresponding size distribution curve and a photo of the AuNP suspensions; (B) UV-vis absorption spectra (top) and zeta potential values (bottom) of AuNPs both after synthesis (AS) or incubation in moDC medium for 1, 24, and 48 h. Data are shown as mean + SD. Dashed line in panel B bottom represents the *Z* potential of FBS.

To do so, we first generated four different batches of monodispersed citrate-stabilized gold NPs with an average diameter of 4.0 ± 0.6 nm, 8.8 ± 1.5 nm, 11.8 ± 0.8 nm and 26.4 ± 3.0 nm, respectively, using two kinetically controlled seeded growth methods. Then, data regarding their *Z* potential, absorption spectra and hydrodynamic diameter after synthesis were acquired *via* laser doppler velocimetry (LDV), transmission electron microscopy (TEM), UV-vis spectroscopy and dynamic light scattering (DLS) and are summarized in Table 1 and Fig. 1A. Interestingly, all the physicochemical properties of the tested nanoparticles were in line with the ones obtained by Bastús *et al.*^26^ and by Piella *et al.*^38^ in their work, highlighting the quality and reliability of the synthetic method used in this study.

Subsequently, since NP aggregation is also known to be a critical factor able to modulate the *in vitro* response of immune cells, such as monocytes and DCs, in terms of NP uptake and cell activation,^39^ we investigated NP stability and behavior in biologically relevant culture medium.

To do so, AuNPs were incubated for 1, 24 and 48 h in moDC medium and analyzed by UV-vis spectroscopy and LDV. Upon data evaluation, we did not detect profile changes of UV-vis spectra (*e.g.* a secondary peak) that would be representative of NP aggregation. This indicates that the 8-, 11-, and 26 nm batches were colloidally stable over the tested period under these conditions. However, the presence of a red-shift in the UV-vis spectra, which increased over time (reaching stable values after 24–48 h), suggests the formation of a protein corona which promotes a change in the local refractive index on the NP surface (Fig. 1B top). This hypothesis was further supported by the data acquired *via* LDV, which show (at 1 h of incubation) an increase in the *Z* potential values comparable to that obtained for FBS (dashed line), suggesting that serum proteins were effectively adsorbed on the NP surface, masking the particle's negative charge (Fig. 1B bottom). Unfortunately, due to the higher scattering intensity of the serum proteins, it was not possible to obtain meaningful data for the 4 nm AuNPs in culture medium.

Taken together, these results suggest that the AuNP batches used in this study are monodispersed, not aggregated, and stable under the tested culture conditions. Thus, we can expect that any potential immunomodulatory effects that these AuNPs exert on human DCs will be due to the activity of single particles rather than their aggregates.

### 3.2 AuNP characterization *via* cell-based assays: cytotoxicity and LPS contamination

LPS is an important component of the bacterial cell wall and, due to its great stability and broad distribution on all surfaces, is frequently encountered as an impurity in pharmaceutical preparations, orthopedic implants and NPs even in the absence of viable bacteria.^40–42^ Because immune cells such as DCs are extremely sensitive to exceedingly low endotoxin concentrations (<20 pg mL^−1^),^41^ testing all NP suspensions for the presence of residual LPS is essential to ensure that possible effects of NPs on immune cells are triggered by the NPs themselves and not by undetected LPS contamination. For this purpose, we performed a cellular TLR4-NF-κB reporter gene assay and a non-cellular EndoLISA test.

Non-cellular assays such as the EndoLISA are based on factors involved in the blood coagulation system of the horseshoe crab *Limulus polyphemus*. These assays take advantage of a coagulation cascade, starting with the binding of LPS to Factor C and ending in the activation of a pro-clotting enzyme.^43^ However, the reliability of non-cellular assays can be affected by an “LPS-masking effect” caused by chelators or detergents, which makes LPS undetectable in Factor C-based assays.^30^ Therefore, we additionally applied an NF-κB reporter gene assay using HEK293 cells transiently transfected with plasmids encoding a functional LPS receptor together with an NF-κB-luciferase reporter plasmid.

**Fig. 2:**
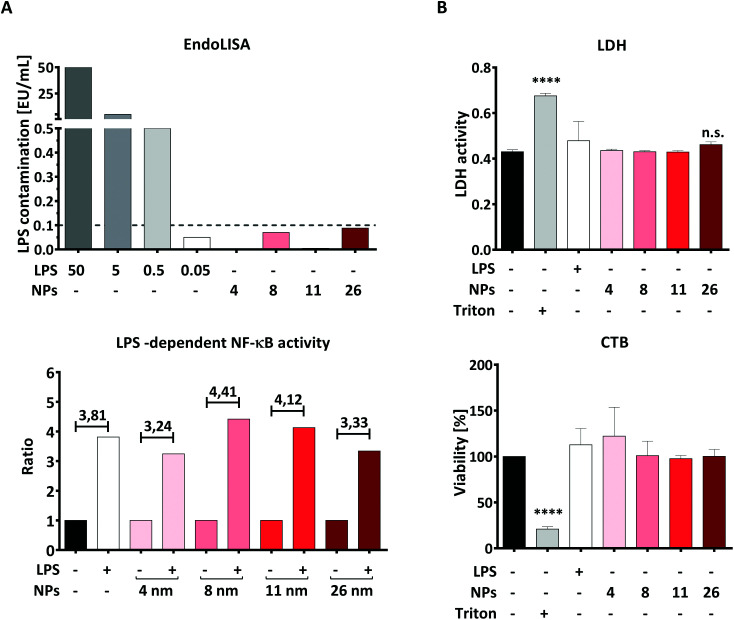
AuNP characterization *via* cell-based assays. Graphs represent (A) assessment of LPS contamination *via* EndoLISA (top) and *via* TLR4/NF-κB-luc reporter gene assay (bottom); here the ratio was calculated between the LPS-containing samples (30 pg mL^−1^ LPS) and the respective controls (AuNPs alone or untreated samples). The dashed line in panel A top represents the minimum amount of LPS capable of activating immune cells. The conversion rate from EU mL^−1^ to ng mL^−1^ is 10 : 1. (B) Cytotoxicity assay based on detection of LDH release and resazurin conversion (CTB) performed on moDCs after 48 h of stimulation with the stated stimuli (5 × 10^11^ NPs per well, 30 ng mL^−1^ LPS) (two individual experiments, *n* = 4). Statistical analysis was performed using repeated-measures ANOVA combined with the Tukey's post test. ****P < 0.0001. Data are shown as mean + SD. CTB, CellTiter-Blue, EU, Endotoxin Unit, LDH, lactate dehydrogenase, LPS, lipopolysaccharide, MFI, mean fluorescence intensity, NF-κB, nuclear factor kappa-light-chain-enhancer of activated B cells, NP, nanoparticle, TLR4, toll-like receptor 4.

Using the EndoLISA assay, we analyzed the endotoxin content in the AuNP stock solutions and found that 8- and 26 nm particles contain a detectable amount of endotoxin, as indicated in ESI Table 1.† It is well documented that even the very low LPS concentration of 0.1 EU mL^−1^ is capable of activating human immune cells, including DCs.^41^ Since the aim of this study is to investigate the effects of AuNPs under inflammatory conditions, we calculated the maximum number of particles for all batches that can be applied to DCs without inducing an immune response induced by contaminating LPS, and found this NP-dose to be equal to 5 × 10^11^ NPs per well (correspondent to 4.16 × 10^11^ NPs per mL). In this context, Fig. 2A top summarizes the estimated endotoxin content in the culture medium after addition of 5 × 10^11^ NPs per well, which is below 0.1 EU mL^−1^ for all AuNP batches used.

To confirm that the endotoxin contamination present in the chosen dose of particles is indeed unable to activate the LPS receptor complex composed of TLR4, MD2 and CD14, we performed an NF-κB-luc reporter gene assay using transiently transfected HEK293 cells. As shown in Fig. 2A, lower panel, the TLR4/MD2/CD14-NF-κB reporter gene assay showed that NF-κB activation is increased in the presence of LPS, both with or without AuNPs, while none of the tested batches was able to activate NF-κB when applied alone at the dose of 5 × 10^11^ NPs per well (Fig. 2A, bottom). Since HEK293 cells expressing the LPS receptor complex are as sensitive to LPS as DCs are,^41^ we conclude that AuNPs applied at a dose of 5 × 10^11^ NPs per well are not sufficient to activate human DCs *via* their residual endotoxin contamination. We thus used this NP concentration for all the following experiments.

The next important step was to quantify, and eventually exclude, NP-induced toxicity, as this can deeply impair the ability of DCs to upregulate surface markers and release soluble mediators, thus possibly masking immunomodulatory properties of NPs. To this end, we performed both lactate dehydrogenase (LDH) and CellTiter-Blue (CTB) assays in moDCs that had been treated with AuNPs for 48 h. The LDH test measures cell death by quantifying LDH, a cytoplasmic enzyme released upon cell membrane lysis, whereas CTB provides information on cellular metabolic activity by assessing the conversion of the substrate resazurin into resorufin by viable cells. As shown in Fig. 2B, no significant cytotoxicity to moDCs was induced by exposing the cells to 5 × 10^11^ NPs per well, which further supports the use of this dose in the following experiments. Interestingly, these results are in line with the comprehensive study by Khlebtsov *et al.*,^44^ who found that AuNPs are generally nontoxic when applied at concentrations lower than 10^12^ NPs per mL. The high background observed in the LDH assay can be explained by LDH naturally present in FBS, which is used as a supplement in the cell culture medium.^45^

### 3.3 26 nm AuNPs decrease the LPS-dependent release of IL-12 and IL-27 in DCs

Monocyte-derived DCs are a subset of DCs which differentiate from monocytes in tissues under inflammatory conditions and play a crucial role in host defense.^46^ These cells can also be generated *in vitro* by culturing monocytes with a cocktail of polarizing cytokines. Therefore, they are an interesting model for investigating nano-immune interactions during acute inflammation.^32^

**Fig. 3:**
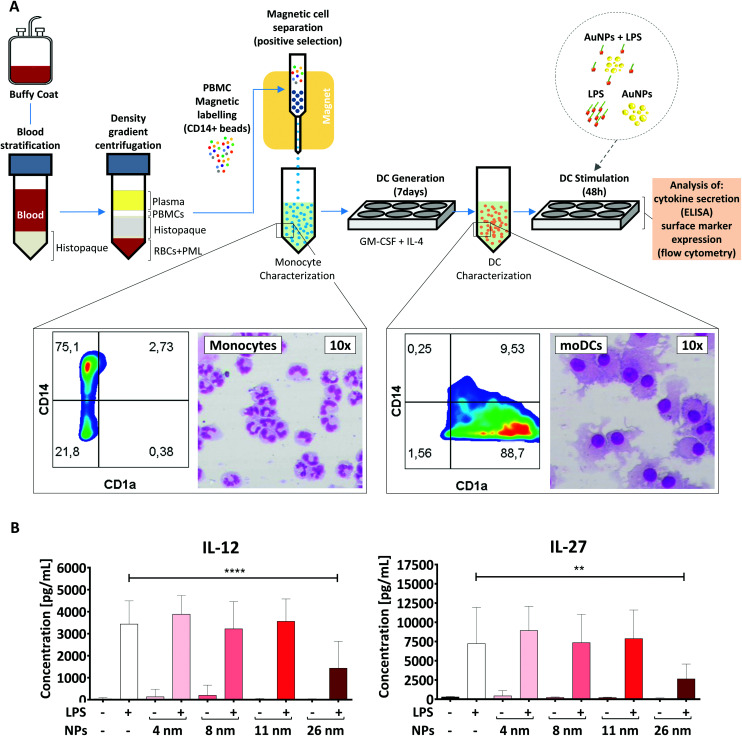
26 nm AuNPs hamper LPS-induced cytokine secretion in moDCs. (A) Schematic representation of the protocol used. Morphological changes occurring during the moDC-differentiation process after MACS-purified monocytes have been incubated with GM-CSF and IL-4 are summarized in the rectangular insets. Monocytes are characterized by a U-shaped nucleus and high CD14 expression, whereas DCs exhibit several cell-protrusions, high CD1a expression and the loss of CD14 on their surface. Cells were stained with Diff-Quik stain and imaged by optical microscopy. (B) To assess the influence of AuNPs on LPS-induced cytokine release, moDCs were cultivated for 48 h with the stated stimuli (5 × 10^11^ NPs per well, 30 ng mL^−1^ LPS) and subsequently supernatant was collected and analyzed for IL-12 and IL-27 secretion *via* ELISA. Statistical analysis was performed using repeated-measures ANOVA combined with Tukey's post test **P < 0.01; ****P < 0.0001 (4 individual experiments, *n* ≥ 7). Data are shown as mean + SD. Significance is shown as indicated. RBC, red blood cell, DC, dendritic cell, LPS, lipopolysaccharide, NP, nanoparticle, PML, polymorphonuclear leukocyte; PBMC, peripheral blood mononuclear cell.

To generate moDCs, we first purified human PBMCs from the blood of healthy donors *via* density gradient centrifugation (Fig. 3A). Subsequently, we incubated these cells with beads coated with anti-CD14 antibodies and extracted a population of CD14^+^ monocytes *via* magnetic cell separation. Monocytes were then differentiated into moDCs in the presence of IL-4 and GM-CSF for 7 days.^31^ To confirm the efficiency of the differentiation protocol, we investigated cell morphology and surface marker expression by differential staining (Diff-Quik) and flow cytometry, respectively. In line with previous studies,^32^ moDCs were characterized by high expression of CD1a, the loss of CD14 and the presence of a round nucleus surrounded by several cytoplasmic projections, and therefore were considered to be fully differentiated. In contrast, monocytes were found to be CD1a^−^, CD14^high^ cells with a lobular nucleus (Fig. 3A).^32^

To assess if AuNPs have an impact on LPS-induced immune responses, the obtained moDCs were incubated for 48 h either with LPS, AuNPs of different sizes (4, 8, 11, and 26 nm), AuNPs plus LPS or left untreated (Fig. 3B). Upon LPS stimulation, DCs are known to become activated and secrete several soluble mediators, which are crucial for Th cell differentiation. As shown in Fig. 3B and ESI Fig. 1,† and in line with previous reports,^29,41,47,48^ the DCs produced high amounts of cytokines (IL-12, IL-27, IL-6, TNF-α) and chemokines (IL-8/CXCL8) in response to LPS stimulation. However, when LPS-treated DCs were co-incubated with 26 nm AuNPs, the secretion of IL-12 and IL-27, which are cytokines involved in promoting the differentiation of Th1 cells,^32,47–50^ was significantly reduced (Fig. 3B). Other cytokines, such as TNF-α, IL-8/CXCL8 and IL-6, which are not clearly associated with Th1 cell development, were not significantly affected by the treatment (ESI Fig. 1†). Interestingly, 4, 8 and 11 nm AuNPs did not impair LPS-dependent DC-activation and none of the tested batches were capable of inducing the release of cytokines in the absence of LPS (Fig. 3B and ESI Fig. 1†).

### 3.4 26 nm AuNPs affect LPS-dependent surface marker expression in moDCs

Another hallmark of DC maturation is the expression of surface markers such as CD86, ILT3 and HLA-DR. Like soluble mediators such as cytokines, these cell-surface proteins play a crucial role in determining the nature and intensity of an immune response. Surface molecules facilitate the physical interaction of DCs and T cells during the process of antigen presentation, whereby HLA-DR, for example, has the function of presenting antigenic peptides to antigen-specific naïve T cells. In combination with signals provided by the costimulatory molecule CD86, which is upregulated upon LPS stimulation, T-cell activation can be induced. In contrast, the co-inhibitory molecule ILT3 counteracts T-cell activation by providing immunoregulatory signals.^51^ Therefore, we assessed the expression of CD86, HLA-DR and ILT3 on moDCs treated with AuNPs/LPS by using flow cytometry.

**Fig. 4:**
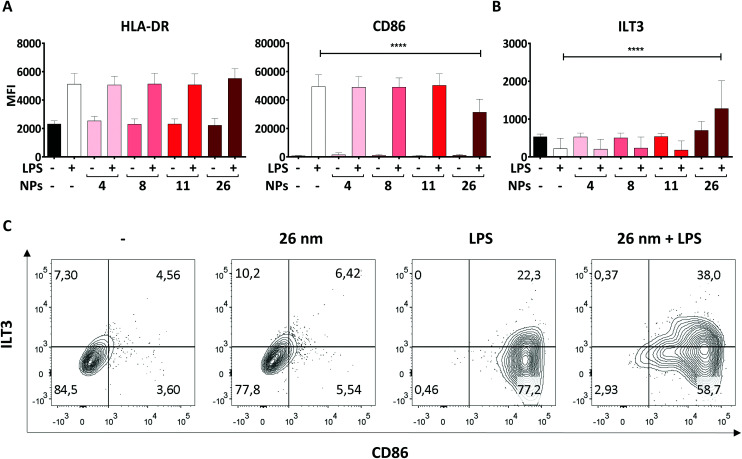
26 nm AuNPs downregulate LPS-induced CD86 expression while promoting ILT3 upregulation. To assess the influence of AuNPs of different sizes on LPS-induced surface marker expression, moDCs were cultivated for 48 h with the stated stimuli (5 × 10^11^ NPs per well, 30 ng mL^−1^ LPS), subsequently the cells were harvested and analyzed *via* flow cytometry. Graphs represent expression in terms of MFI of (A) pro-inflammatory markers and (B) anti-inflammatory markers on moDCs after stimulation. (C) Dot-plots depicting ILT3 and CD86 expression in control DCs and DCs treated with LPS, 26 nm AuNPs, or 26 nm AuNPs/LPS. Samples are from of one representative donor. Statistical analysis was performed using repeated-measures ANOVA combined with the Tukey's post test. ****P < 0.0001 (three individual experiments, *n* = 6). Data are shown as mean + SD. LPS, lipopolysaccharide, MFI, mean fluorescence intensity, NP, nanoparticle.

In line with previous studies, LPS promoted significant upregulation of both HLA-DR and CD86 expression on the DC surface in comparison to untreated control cells (Fig. 4A).^29,52^ Interestingly, 4, 8, and 11 nm AuNPs did not modulate the DC response to LPS (Fig. 4A). However, treatment with 26 nm AuNPs/LPS caused significant downregulation of CD86 (Fig. 4A), accompanied by robust upregulation of ILT3 (Fig. 4B), while HLA-DR remained unchanged. Fig. 4C shows the relative expression of CD86 and ILT3 on cells of one representative donor.

To exclude that our findings are batch-specific, we repeated all experiments using a batch of fully characterized 25 nm AuNPs produced as described for the first batch and obtained similar results (ESI Table 2 and Fig. 2†).

Taken together, the results described in Section 3.3 and 3.4 demonstrate that 26 nm AuNPs, but not smaller AuNPs, exert regulatory effects capable of impairing LPS-dependent DC maturation, potentially affecting the capacity of DCs to promote T-cell activation. Interestingly, AuNPs exerted these immunomodulatory effects only in the presence of a concomitant microbial stimulus (LPS). The fact that immunomodulating effects of AuNPs on DCs are rare is well documented in the literature^53–55^ and explains why AuNPs are generally considered to be safe and suitable for biomedical applications. In the context of ongoing inflammation, however, we found that our largest AuNPs did hamper DC activation, promoting the acquisition of a semi-mature phenotype characterized by reduced expression of the activation marker CD86, diminished secretion of the pro-inflammatory cytokines IL-12 and IL-27, and concomitant upregulation of the anti-inflammatory receptor ILT3. This phenotype differs substantially from that commonly induced by LPS^10^ or other bacterial stimuli,^56^ suggesting that the activation of anti-bacterial Th1 cells, which are usually generated upon LPS stimulation, and whose differentiation relies on CD86, IL-12 and IL-27 secretion,^50,57,58^ may be significantly impaired by the application of 26 nm AuNPs. A semi-mature phenotype is a typical feature of tolerogenic DC (tDCs). tDCs, by presenting antigens in the context of reduced costimulatory capacity (CD86^low^) and a significantly altered cytokine profile, can promote tolerance and T-cell anergy, which are important mechanisms for eliminating alloreactive T cells and regulating the intensity and duration of inflammatory immune responses.^59^ In this scenario, ILT3, which is a characteristic marker of tDCs,^60^ plays a crucial role in the suppressor capacity of T cells and induces T-cell anergy.^61^

It is worth noting that, to our knowledge, this is the first study to report that citrate-stabilized AuNPs can affect IL-27 and ILT3 production. Effects of AuNPs on CD86 and IL-12 were previously reported by Villiers *et al.* and Tomić *et al.*, who observed similar immunomodulatory effects.^54,55^ However, in our study, the effects were induced exclusively by 26 nm AuNPs (and not 10 nm AuNPs). Moreover, we saw no evidence of effects on HLA-DR expression. This discrepancy might be due to several factors, including the doses of the stimulants, NP purity and NP concentrations.

Interestingly, in our study, only factors involved in Th1 polarization (CD86, IL-12, IL-27) were affected by AuNP treatment, while the ability of DCs to express HLA-DR, IL-8/CXCL8, TNF-α and IL-6 was not impaired. These findings suggest that DCs may retain their ability to promote antigen presentation (HLA-DR), neutrophil recruitment (IL-8/CXCL8),^62^ and Th17 differentiation (IL-6).^11^

### 3.5 Ultrastructure of the endosomal system and intracellular localization of NPs

**Fig. 5:**
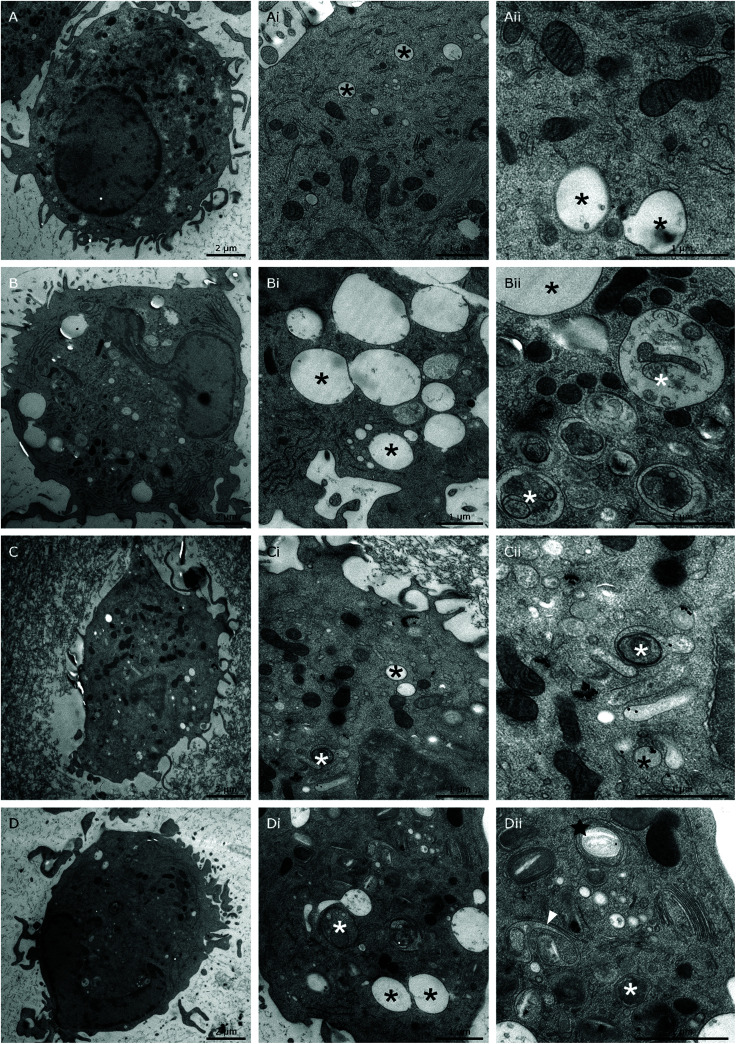
AuNP intracellular localization. To assess if 26 nm AuNPs are phagocytosed by DCs and if they can change the cellular phenotype at the ultrastructural level, moDCs were treated with the following stimuli and subsequently prepared for TEM inspection. (A-Aii) Untreated control, (B-Bii) LPS control; (C-Cii) 26 nm AuNPs; (D-Dii) 26 nm AuNPs + LPS. Black asterisks, endosomes; white asterisks, autophagic structures; black star, class E-like compartment; white arrowheads, “frustrated” MVBs.

In our attempt to understand the molecular mechanisms underlying the effects of 26 nm AuNPs, we performed uptake studies and ultrastructure analysis of our untreated (Fig. 5A-Aii), LPS-treated (Fig. 5B-Bii), AuNP-treated (Fig. 5Cii and ESI Fig. 3A, B†) and AuNP/LPS-treated DCs (Fig. 5D-Dii and ESI Fig. 3C, D†) *via* transmission electron microscopy (TEM) after cryofixation. The obtained data show that the internal structure and, more precisely, the endosomal system of LPS-activated DCs (Fig. 5B-Bii) differ substantially from those of untreated control cells (Fig. 5A-Aii).^63^ In particular, LPS induced an increase in the size (up to 2 μm) of electron-lucent late endosomes,^64^ some of which are characterized by the presence of intraluminal “buds” representing the early steps of formation of intraluminal vesicles (ILVs, black asterisks),^65^ which are indicative of the endosomal transition to multivesicular bodies (MVBs).^66^ These endosomes were often accompanied by the appearance of autophagosomes (with intact cytoplasm) and autophagic compartments (partly degraded material) in large numbers (white asterisks).^64^

When DCs were treated with AuNPs instead, a clear uptake/internalization was visible, both in the absence (Fig. 5C-Cii and ESI Fig. 3A, B†) or presence of LPS (Fig. 5D-Dii and ESI Fig. 3C, D†). In all the examined images, AuNPs were always found enclosed within vesicles, and when the DCs were treated exclusively with NPs, the ultrastructure of the DC endosomal compartment was not significantly different from that of control cells. Interestingly, in this case, NPs were found to be mainly localized in tubule-vesicular structures of the endosomal system (Fig. 5Cii and ESI Fig. 3A, B†) and occasionally in late endosomes containing ILVs (black arrowhead) (ESI Fig. 3A†). Overall, the tubule-vesicular structures where composed of both electron-light (low buoyant density) and electron-dense (high buoyant density) vesicles, with the latter resembling classic lysosomes.^67^

In contrast, when DCs were co-exposed to LPS and AuNPs (Fig. 5D-Dii), we observed dramatic changes in cell ultrastructure and NP localization in comparison to both LPS-treated and AuNP-treated DCs.

In the doubly treated cells, there was reduced formation of large electron-lucent late endosomes, and the NPs were found to be mainly segregated within autophagic compartments. Interestingly, only the combination treatment promoted the formation of multilamellar cisternae organized in stacks (black stars) (Fig. 5Dii) or U-shaped invaginated vesicles observed in cross-section (white arrowheads) (Fig. 5Dii and ESI Fig. 3C, D†) resembling the E class compartments described by Russel *et al.* and other authors, which are considered a form of “stressed” MVBs.^65,68–70^

The formation of class E compartments and “stressed” multivesicular bodies is strongly dependent on the activity of the ESCRT machinery. When ESCRT is dysfunctional, endosomes and MVBs stop their correct maturation process, do not produce ILVs, become “frustrated” and cluster to form class E compartments.^68,69,71^ The ESCRT machinery plays a role not only in MVB maturation, but also in different steps of the autophagic pathway. In fact, the depletion of ESCRT proteins in cells is commonly associated with the accumulation of autophagosomes, similar to what we observed in our double-treated samples.^72^ Interestingly, AuNPs were shown by Ma *et al.* to be able to block autophagy and alter the activity of the endosomal compartment by increasing the internal pH of these vesicles and *de facto* blocking their fusion with lysosomes.^73^ In line with these findings, ESCRT components have been found in fungi to be part of a pH-sensing complex which, under alkaline conditions, changes its localization from the endosomal compartment to the plasma membrane,^74^ suggesting that pH changes might indeed play a crucial role in the functionality of the DC endocytic compartment.

This altered ultrastructural phenotype might partially explain our finding at the cytokine level. In fact, LPS signaling is known to rely on two different pathways: the MyD88-dependent cytosolic pathway, and the TRAF-TRIF-dependent pathway, which relies on receptor endocytosis.^4,66,75^ Once activated, both pathways promote the expression of several genes linked to inflammation. However, while it has been reported that TNF-α and IL-6 are mainly regulated by MyD88, IL-12 secretion depends on signaling along both pathways, suggesting that its production might be affected by NP-mediated changes in receptor endocytosis and trafficking toward different endocytic compartments such as the endosomes or MVBs.^66,76–78^ Additionally, it has been suggested by Gangloff that endosomal pH and curvature (which is size-dependent) may also play a role in the capacity of TLR4 to recruit TRAM and activate the downstream signaling pathways.^75^

Therefore, we propose that deviations in the endocytic pathway may partially explain the observed effect of AuNPs on DC activation; however, further studies are needed to confirm this hypothesis.

### 3.6 26 nm-treated DCs are less proficient at promoting Th1 activation

**Fig. 6:**
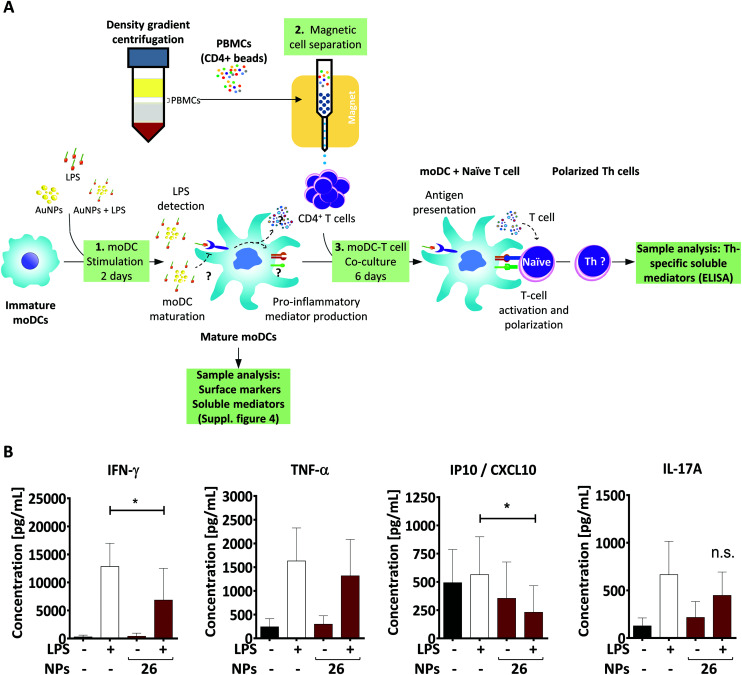
26 nm AuNPs downregulate the expression of Th1-related cytokines. (A) To assess the influence of 26 nm AuNPs on the ability of moDCs to induce T-cell polarization, especially toward the Th1 phenotype, pre-treated moDCs (A1) were co-cultured with allogenic CD4^+^ T cells, obtained *via* magnetic cell separation (A2), for 6 days (A3). Afterwards, supernatants were analyzed by ELISA or LegendPLEX to investigate the secretion of Th-specific soluble mediators, which are shown in (B). Statistical analysis was performed using repeated-measures ANOVA combined with the Tukey's post test (five individual experiments, *n* ≥ 8). Data are shown as mean + SD. *P < 0.05. AuNP, gold nanoparticle; LPS, lipopolysaccharide; NP, nanoparticle; n.s., not significant; PBMC, peripheral blood mononuclear cell; Th, T helper cell.

Upon microbial infection, DCs quickly initiate adaptive immune responses by activating T lymphocytes. For this reason, it is important to assess whether the changes we detected in the DC phenotype, including the expression of surface molecules, cytokine secretion, formation of endocytic structures and intracellular localization of AuNPs, affect the DC's ability to induce T-cell activation. A well-accepted model for studying such aspects is the allogeneic co-culture system. In this study, we first pre-treated moDCs for 48 h and then assessed their stimulatory capacity by co-culturing them with allogeneic CD4^+^ Th-cells for 6 days (Fig. 6A). During this period, DCs provide important signals to Th cells, which instruct the latter to secrete cytokines indicative of their specific function.

To confirm the quality of the APCs used for the co-culture experiment, the activation state of moDCs was fully characterized by flow cytometry and enzyme-linked immunosorbent assay (ELISA) prior to co-incubation with T cells (ESI Fig. 4†).

As mentioned above, LPS-stimulated moDCs would be expected to induce a specific T-cell subpopulation known as Th1 cells, which mainly secrete IFN-γ and IFN-γ-related factors.^11,79^ Consistent with this, LPS-treated moDCs promoted the release of IFN-γ as well as the IFN-γ-inducible protein CXCL10 in corresponding Th cells (Fig. 6B). However, the production of these factors was significantly lower in Th cells co-cultured with 26 nm AuNP-treated LPS-stimulated DCs, whereas other cytokines which are not associated with Th1 activation (*e.g.*, TNF-α and IL-17) were not affected by 26 nm AuNPs.

In fact, the secretion of IL-27 by DCs is of crucial importance for the initial commitment of naïve T cells to the Th1 lineage, because this cytokine is able to promote the induction of the Th1-specific transcription profile, which is subsequently stabilized by sustained IL-12 signaling.^32,47–50,80^ Since IL-27 was significantly downregulated by 26 nm AuNPs (Fig. 3B) and the IL-27/IL-12-induced transcription profile is also directly responsible for the secretion of IFN-γ in Th1 cells, it was not surprising to find IFN-γ significantly reduced in T cells incubated with LPS/AuNP-treated DCs. In accordance with this, we also observed a lower secretion of the IFN-γ-dependent chemokine CXCL10, which is known to attract many cell types to sites of infection.^79^

Because Th1 cells and their lead cytokine IFN-γ play a crucial role in macrophage activation and the recruitment of cytotoxic cells, the observed loss of Th1 cells might have a negative effect on both pathogen clearance and elimination of cancerous cells.^81–85^ However, Th1 cells also seem to be involved in the pathogenesis of autoimmune diseases such as rheumatoid arthritis,^86^ which, interestingly, was treated in the past using gold-based therapeutics. This suggests that the observed effects of AuNPs on Th1 differentiation might be advantageous in the presence of autoimmune diseases, but potentially harmful in the context of pathogen clearance.

Our observation that AuNPs do not alter the maturation or activation of DCs in the absence of a concurrent microbial stimulus is consistent with a previous study by Malachin *et al.*, who showed that silica NPs (SiNPs) are able to potentiate the ability of DCs to activate T cells in response to PAMPs, while SiNPs alone were unable to activate DCs, unless administered in combination with microbial components.^87^ Taken together, these studies further support the importance of investigating NP immunomodulatory properties during ongoing immune responses and not only under homeostatic conditions.

### 3.7 26 nm AuNPs affect Th1 and TCMc numbers and proliferation

**Fig. 7:**
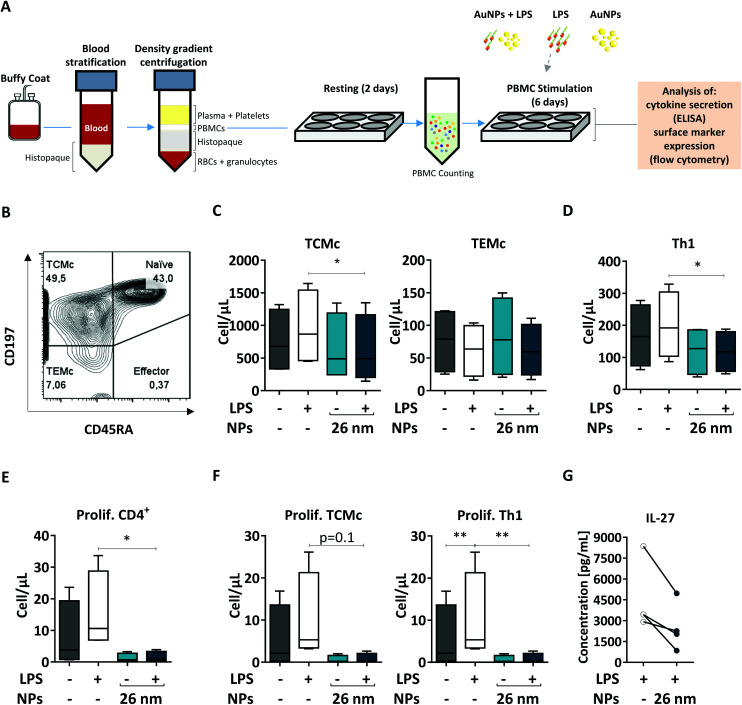
26 nm AuNPs downregulate total numbers of TCMc and Th1 cells and T-cell proliferation. To assess the activity of 26 nm AuNPs in a more realistic model, human PBMCs were incubated, after resting, for 6 days with the stated stimuli (5 × 10^11^ NPs per well, 30 ng mL^−1^ LPS), and subsequently the supernatants were analyzed *via* ELISA and the cells were analyzed *via* flow cytometry (two individual experiments, *n* = 4). (A) Graphic summary of the protocol. (B) Representative dot plot depicting the distribution of the T cells within the different CD4+ T cell populations (Naïve, Effector, TEMc, TCMc) upon PBMC stimulation with 26 nm AuNPs ± LPS. Untreated PBMCs serve as a control. Graphs showing (C) total memory (TCMc and TEMc), (D) total Th1, (E) total proliferating CD4+, and (F) proliferating TCMc and Th1 cells expressed in terms of cells per μL. (G) IL-27 secretion from PBMCs. Statistical analysis was performed using repeated-measures ANOVA combined with the Tukey's post test. *P < 0.05; **P < 0.005. Data are shown as mean + SD. DC, dendritic cell; LPS, lipopolysaccharide; NP, nanoparticle; PBMC, peripheral blood mononuclear cell; TCMc, T central memory cell; TEMc, T effector memory cell; Prolif. proliferating.

When NPs are used for medical applications, they are often injected intravenously into the bloodstream, where they come into contact with a pool of immune cells called peripheral blood mononuclear cells (PBMCs). Within this pool, both DCs and T cells are present; therefore, blood-derived PBMCs are considered a good model to investigate the effects of NMs on homologous cell cultures.^33^ Here, we first extracted human PBMCs from the blood of healthy donors, and incubated them for 6 days in the presence of the respective stimuli (26 nm AuNPs, LPS, and LPS plus 26 nm AuNPs). Finally, we assessed the effects of LPS and 26 nm AuNPs on the activation of both Th1 and memory cells *via* flow cytometry (Fig. 7A) using an advanced 13-color flow cytometry panel (the gating strategy is summarized in ESI Fig. 5†).

In human blood, we find mainly two types of memory cells: central memory T cells (TCMc) and effector memory T cells (TEMc).^33^ These populations can be distinguished based on the expression of distinct homing receptors which grant them different migration capacities. TCMc express CD197, which allows their recruitment to lymphatic tissues, whereas TEMc express mainly markers that direct them to the periphery. Memory cells do not express the surface marker CD45RA and therefore can be easily distinguished *via* flow cytometry from CD45RA^+^ naïve and effector cells.^88^

As shown in Fig. 7B, by using these markers, we could demonstrate that memory T cells (including TCMc and TEMc) represent the majority of the CD4^+^ Th cell populations in PBMCs,^33^ with TCMc being the most abundant subset. Upon treatment, memory cells responded differently to stimulation. We observed an increase in the total number of TCMc, but not of TEMc, in LPS-treated samples. Interestingly, this expansion was significantly hampered by 26 nm AuNPs in TCMc, while there was no clear effect on LPS-stimulated TEMc (Fig. 7C).

We then investigated the amount of Th1 cells present in our samples, as they are the subset most responsive to LPS and other bacterial products. As shown in Fig. 7D, 26 nm AuNPs caused a decrease in LPS-dependent cell expansion, significantly lowering the total number of Th1 cells. A reduced number of cells is often linked to limited proliferation capacity in the tested subpopulations. We therefore labeled our samples with antibodies directed against the proliferation marker Ki-67 and found that LPS-dependent proliferation of total, TCMc and Th1 CD4^+^ lymphocytes was indeed significantly reduced by 26 nm AuNPs (Fig. 7E and F).

Several soluble mediators, such as IL-27, are known to regulate the ability of these cells to proliferate. Thus, we tested our cell supernatant and found a positive correlation between the loss of proliferative potential and reduced IL-27 secretion (Fig. 7G). This is of particular importance because this cytokine also plays a crucial role in Th1 differentiation.^89,90^

Taken together, these experiment on autologous cells further support the findings generated in the allogeneic co-culture on Th1 activation both in terms of number and proliferative potential of this population. Additionally, utilizing flow cytometry, we proved that the presence of AuNPs diminished not only Th1 cells but also the ability of TCMc to proliferate in response to LPS stimulation. This fact may be linked to the reduced costimulatory capacity of DCs characterized by both the upregulation of ILT3, which promotes T-cell anergy,^61^ and the attenuated expression of CD86 as well as the reduced secretion of IL-27 and IL-12. Interestingly, only central and not effector memory cells reacted to LPS and were consequently affected by AuNPs. This fact might depend on several differences that characterize these two subsets. For example: (1) TCMc are more prone to proliferate and might therefore be more sensitive to reduced production of IL-27; (2) TCMc seem to be characterized by a lower activation threshold than TEMc, which might make them more sensitive to LPS stimulation; and finally, (3) TCMc tend to interact more with professional APCs in comparison to TEMc, and therefore might be more impacted by the presence of tDCs.^8^ These results suggest that AuNPs negatively affect not only Th1 functions, but also the activation and proliferation of lymphocytes within the central memory compartment, potentially affecting the outcome of immune responses in the long term.

## 4. Conclusions

Here we provided experimental evidence to show that, in the absence of pro-inflammatory stimuli, AuNPs of different sizes can be considered to be immunologically safe, since they show neither immunomodulatory properties nor the ability to significantly affect, upon internalization, the ultrastructure of DCs up to a concentration of 5 × 10^11^ NPs per well. However, when 26 nm AuNPs (but not smaller ones) are combined with LPS, they can direct DCs toward a tolerogenic-like phenotype (CD86^low^, IL-12^low^, IL-27^low^, ILT3^high^, class E compartments^+^), which is less proficient in promoting LPS-dependent Th1 activation and TCMc proliferation, both in allogeneic co-culture systems and in autologous PBMC culture settings. We conclude that especially under pro-inflammatory conditions, 26 nm AuNPs may have immunomodulatory capacities. For further studies, we would therefore recommend investigating the effects of NMs in both non-activated as well as activated immune cells to better understand their immunomodulatory functions and to predict potential effects of NMs on ongoing immune responses.

Taken together, these findings further support the idea that AuNPs are safe under homeostatic conditions; however, particular care should be taken when deploying them in the course of ongoing immune responses.

## Author contributions

Conceptualization: S.M., F.B., A.D., D.D, V.P., U.L., J.H.H. Investigation: S.M., F.B., A.P., P.S., A.A., R.W., T.V. Visualization: S.M., F.B., P.S. Writing – original draft: S.M., D.D., P.S., J.HH. Writing – review & editing: S.M., F.B., P.S., A.D., D.D., J.HH. Supervision: A.D., D.D., J.HH. Funding acquisition: A.D., D.D., V.P., J.HH.

## Conflicts of interest

There are no conflicts to declare.

## Supplementary Material

NR-013-D0NR09153G-s001
