# Supplementary material for: Gold nanoparticles (AuNPs) impair LPS-driven immune responses by promoting a tolerogenic-like dendritic cell phenotype with altered endosomal structures
Source: Nanoscale. 2021 Apr 15;13(16):7648–66. doi: 10.1039/d0nr09153g (PMC8087175; doi:10.1039/d0nr09153g)
Supplement: NR-013-D0NR09153G-s001 [file NR-013-D0NR09153G-s001.pdf]

Electronic Supplementary Material (ESI) for Nanoscale.
This journal is © The Royal Society of Chemistry 2021

Electronic Supplementary Material (ESI)

Gold Nanoparticles (AuNPs) Impair LPS-driven Immune Responses by Promoting a Tolerogenic-like Dendritic Cell Phenotype with Altered Endosomal Structures

*Sara Michelini, Francesco Barbero, Alessandra Prinelli, Philip Steiner, Richard Weiss, Thomas Verwanger, Ancuela Andosh, Ursula Lütz-Meindl, Victor F. Puntes, Damjana Drobne, Albert Duschl, Jutta Horejs-Hoeck**

Supplementary Table 1. Detection of LPS contamination via EndoLISA assay.

Batch Name	LPS Contamination
4	n.d.
8	0.840 EU/mL
11	0.057 EU/mL
26	0.360 EU/mL

Table summarizing the endotoxin levels detected via EndoLISA in the NP stock solutions after synthesis. n.d., not detectable (below detection limit).

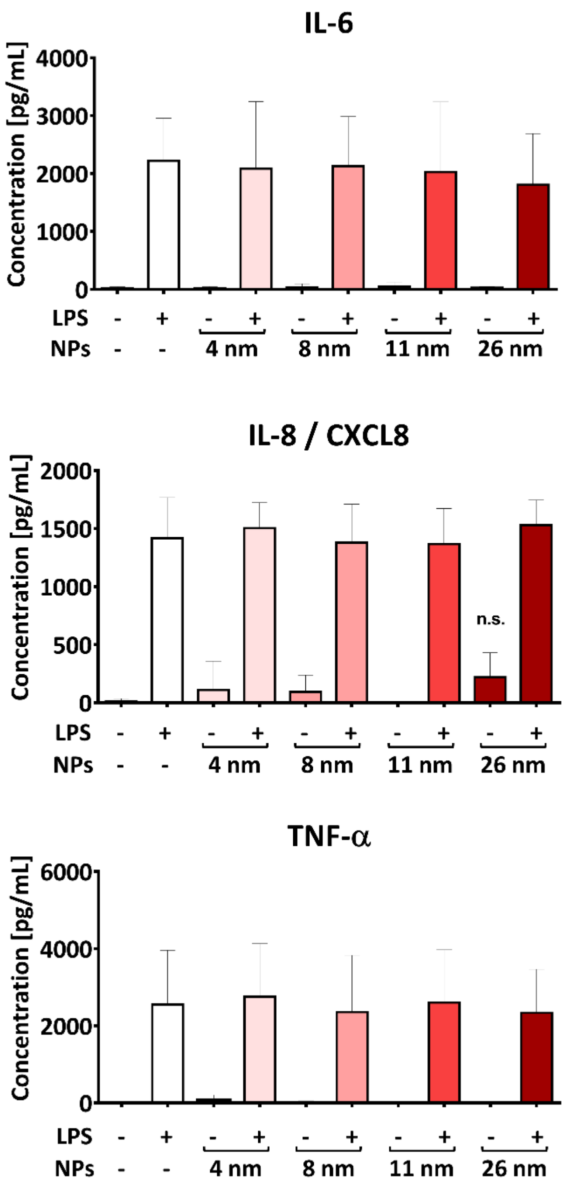

Supplementary Figure 1. Effects of AuNPs on LPS-dependent cytokine secretion by DCs. To assess the influence of AuNPs of different sizes on LPS-induced cytokine release, moDCs from at least 7 individual donors were cultivated for 48 h with the stated stimuli, and subsequently supernatants were collected and analyzed by ELISA (four individual experiments). Significance is shown as indicated. LPS, lipopolysaccharide, n.s., not significant, NP, nanoparticle.

Supplementary Table 2. Summary of 25-nm AuNP post-synthesis characterization**.**

Characteristics	Batch
Name	25
Material	Au
Stabilizer	Sodium Citrate
Diameter by STEM [nm]	25.0 ± 3.0
Hydrodynamic Diameter [nm]	32.8 ± 10.4
Absorption peak [nm]	524
Z potential [mV]	-31.3 ± 0.6
Concentration [NPs/mL]	2.4×10^{12}
Au Concentration [mᴍ]	0.19
Au Concentration [mg/mL]	0.37
Total Surface Area [nm/mL]	4.7×10^{15}

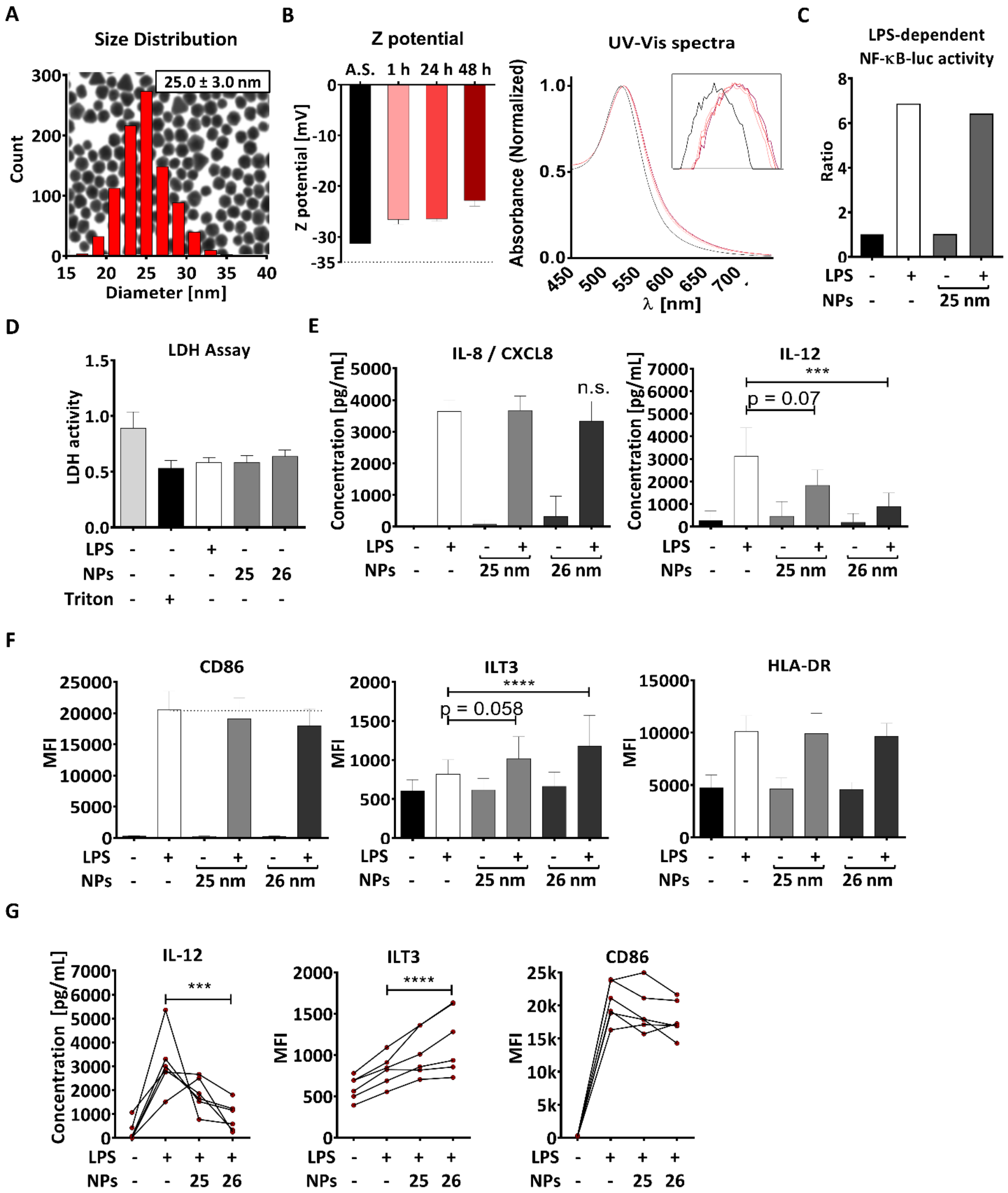

Supplementary Figure 2. 25-nm AuNPs induce effects similar to the 26-nm AuNPs. To assess the replicability of the obtained data, we repeated the experiments shown in Figure 1 to 4 using a different batch of fully characterized AuNPs of similar size, i.e., 25-nm AuNPs. Graphs represent **A-D)** Characterization of 25-nm AuNPs post-synthesis and in media. The concentration of LPS used in **C)** is 30 pg/mL. **E)** Cytokine release by moDCs after 48 h of stimulation. **F)** Surface expression of pro- and anti-inflammatory markers on moDCs after 48 h of stimulation. **G)** Detailed expression levels of markers of interest donor by donor (three individual experiments, *n*=6). Statistical analysis was performed using repeated-measures ANOVA combined with the Tukey's post test. ***P < 0.001; ****P < 0.0001. Data are shown as mean + SD. LPS, lipopolysaccharide, MFI, mean fluorescence intensity.

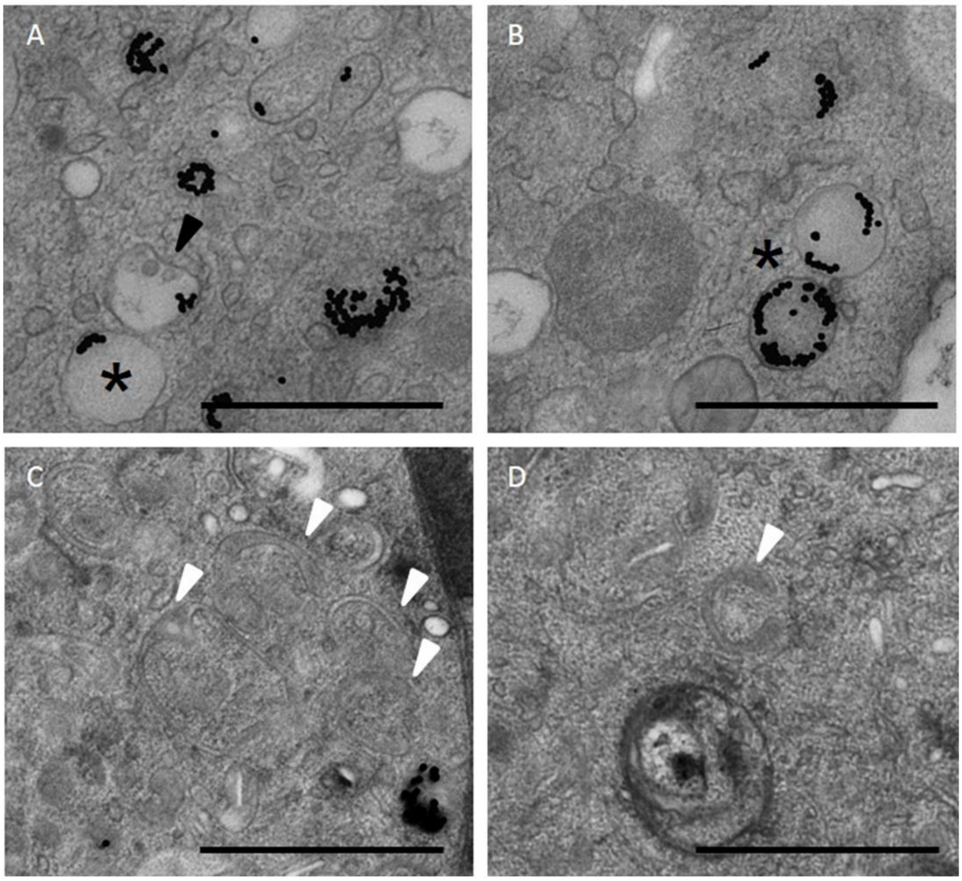

Supplementary Figure 3. 26-nm AuNP intracellular localization. To assess if 26-nm AuNPs are phagocytosed by DCs and if they can change their phenotype at the ultrastructural level, moDCs were treated with the following stimuli and subsequently prepared for TEM inspection. **A-B)** 26-nm AuNP-treated DCs, **C-D)** 26-nm AuNP + LPS-treated DCs. White arrowheads, “frustrated” MVBs; black arrowhead, late endosome (MVBs with ILVs). Scalebar 1 µm.

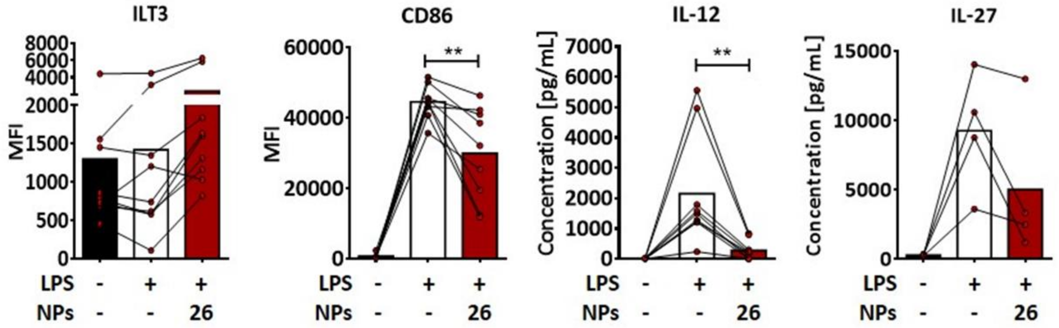

Supplementary Figure 4. Characterization of moDCs used in the co-culture experiment. To assess if the AuNPs induced the same effects on moDCs used in the co-culture experiment, moDCs stimulated for 48 h were tested for cytokine release and surface marker expression via ELISA and flow cytometry, respectively. Graphs represent cytokine release and surface marker expression by moDCs stimulated for 48 h with the indicated stimuli, donor by donor. Statistical analysis was performed using repeated-measures ANOVA combined with the Tukey's post test. **$P < 0.01$. Significance is shown as indicated. LPS, lipopolysaccharide.

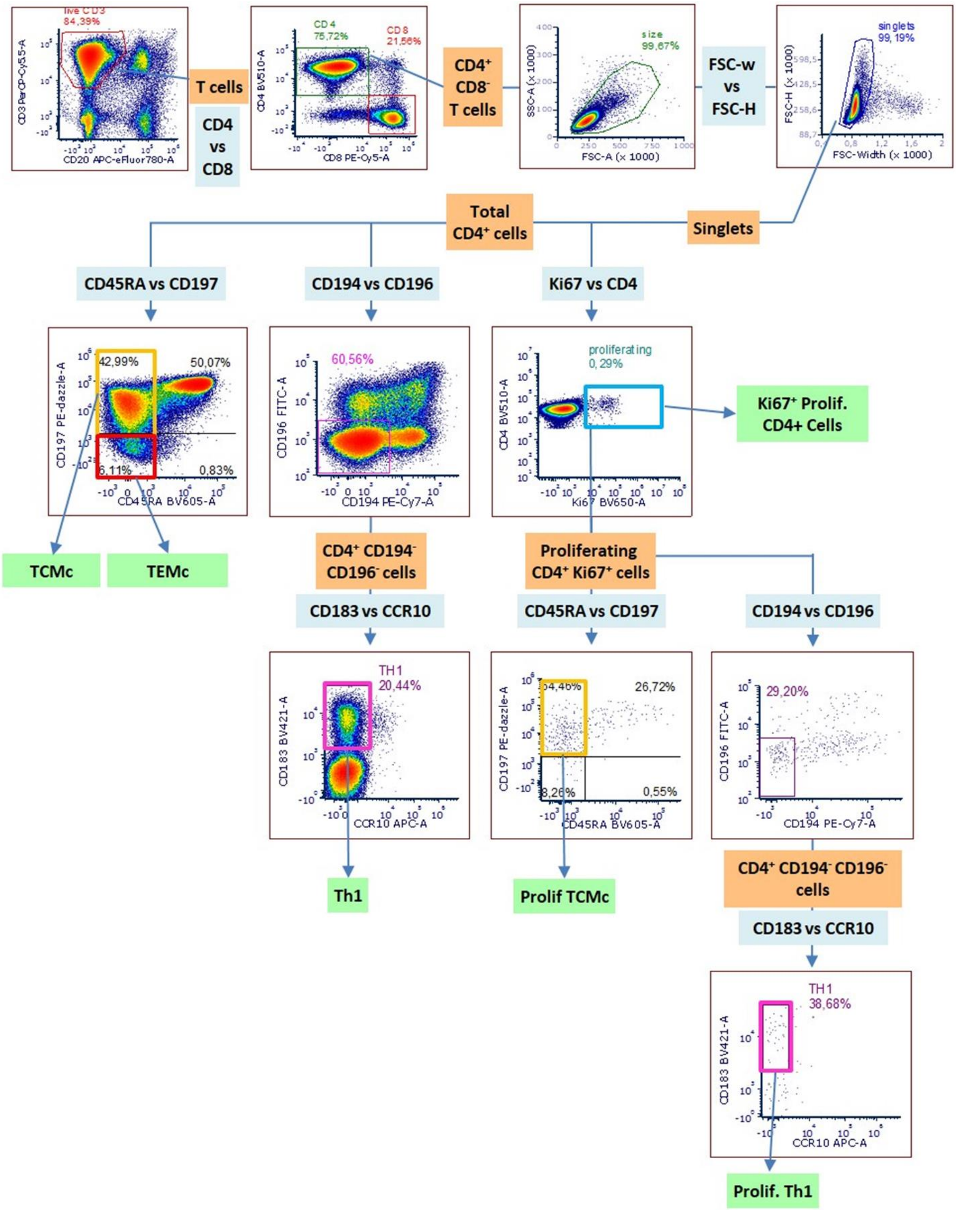

Supplementary Figure 5. 13-color flow cytometry panel gating strategy. The gating strategy applied for analyzing the PBMC response to AuNP treatment. Orange boxes indicate which population is gated to proceed with the analysis, whereas the blue boxes show the markers used for plotting the subsequent sub-population. Green boxes show the populations from which the graphs in **Figure 7** were generated**.** The acquired data were expressed in cells/μL. TCMc, T central memory cell; TEMc, T effector memory cell; Th1, T-helper 1 cell, Prolif, proliferating.
